# Methyl-Cytosine-Driven Structural Changes Enhance Adduction Kinetics of an Exon 7 fragment of the p53 Gene

**DOI:** 10.1038/srep40890

**Published:** 2017-01-19

**Authors:** Spundana Malla, Karteek Kadimisetty, You-Jun Fu, Dharamainder Choudhary, John B. Schenkman, James F. Rusling

**Affiliations:** 1 University of Connecticut, Department of Chemistry, Storrs, CT 06269, USA; 2 UConn, UConn Health, Department of Surgery and Neag Cancer Center, Farmington, CT 06032, USA; 3 Professor Emeritus, UConn Health, Farmington, CT 06269, USA; 4 University of Connecticut, Institute of Material Science, Storrs, CT 06269, USA; 5 School of Chemistry, National University of Ireland at Galway, School of Chemistry, Ireland

## Abstract

Methylation of cytosine (C) at C-phosphate-guanine (CpG) sites enhances reactivity of DNA towards electrophiles. Mutations at CpG sites on the p53 tumor suppressor gene that can result from these adductions are in turn correlated with specific cancers. Here we describe the first restriction-enzyme-assisted LC-MS/MS sequencing study of the influence of methyl cytosines (MeC) on kinetics of p53 gene adduction by model metabolite benzo[a]pyrene-7,8-dihydrodiol-9,10-epoxide (BPDE), using methodology applicable to correlate gene damage sites for drug and pollutant metabolites with mutation sites. This method allows direct kinetic measurements by LC-MS/MS sequencing for oligonucleotides longer than 20 base pairs (bp). We used MeC and non-MeC (C) versions of a 32 bp exon 7 fragment of the p53 gene. Methylation of 19 cytosines increased the rate constant 3-fold for adduction on G at the major reactive CpG in codon 248 vs. the non-MeC fragment. Rate constants for non-CpG codons 244 and 243 were not influenced significantly by MeC. Conformational and hydrophobicity changes in the MeC-p53 exon 7 fragment revealed by CD spectra and molecular modeling increase the BPDE binding constant to G in codon 248 consistent with a pathway in which preceding reactant binding greatly facilitates the rate of covalent S_N_2 coupling.

The p53 (or TP53) tumor suppressor gene codes for p53 protein that regulates cell division, cell death, genomic stability and suppresses cancer. P53 gene mutations occur in over 50% of human cancers^1^, and are located selectively in exons 5–8, and alter the p53 protein so as to minimize cancer protection. Furthermore, mutation sites are clearly correlated with specific cancers^2^,^3^, so that prediction of the most reactive p53 codons toward new chemicals and their metabolites could be a valuable tool to aid in toxicity and cancer prediction. Within the p53 gene, 42 CpG sites are methylcytosines (MeC)^4^,^5^, and are the most frequently mutated codons or *hot spots*^6^,^7^. Non-CpG MeC related to GC > AT transitions are known in lung, and head and neck cancers^2^,^8^.

Reactants such as benzo[a]pyrene-7,8-dihydrodiol-9,10-epoxide (BPDE, Fig. 1a), 7,12-dimethylbenz(a)anthracene diol epoxide, acrolein, and mitocin C, are more reactive towards MeCpG sites on the genes^4^,^5^. Limited repair of these reacted sites contributes to their role as mutational hot spots. Polyaromatic hydrocarbon (PAHs) metabolites preferentially react with MeCpG sites^9^. E.g., benzo[a]pyrene is metabolized to BPDE by a sequential pathway involving cytochrome P450s and epoxide hydrolase^10^,^11^, and reacts with DNA in S_N_2 reactions to form nucleobase adducts that lead to mutations^12^. Previous studies of alkylated CpGs on DNA exon 5 analogs using [^15^N_3_,^13^C_1_]-labeled guanines and oligonucleotide hydrolysis revealed 2-3 fold increases in yields of BPDE adduction for MeCs^13^. MeCpG’s also enhanced DNA-acrolein adduction 2-fold^14^,^15^, A 21-base pair (bp) oligonucleotide with a CpG site gave MeC-dependent voltammetry suggesting enhanced BPDE reactivity^16^. A 14 bp oligonucleotide with four MeCpG sites gave 3–4 fold increased adduction yields compared to unmethylated CpG^17^. In the 1990s, Geacintov measured strong non-covalent binding between BPDE and ds-poly(dG-dC).(dG-dC) oligonucleotides and proposed enhanced subsequent coupling with MeCs related to stronger binding in a preceding step^18^,^19^. This model was supported by studies of alkyl-C exon 5 analogs^13^.

We recently reported a restriction-enzyme-assisted LC-MS/MS DNA sequencing method that extends reactive codon measurements to ds-DNA lengths longer than 20-bp^20^. For a 32 bp exon 7 p53 duplex fragment, we found adduct yields for reaction with BPDE in the order of codons 248 > 243 > 244. In the present paper, we adapt this method for the first time to elucidate relationships between codon-specific reaction kinetics with metabolites, and evaluate the influence of subtle but important MeC-related p53 gene structure changes on reaction kinetics. This approach enabling studies of longer nucleotides than have been previously possible is amenable to uncover the influence of important structural changes relevant to the reactivity of the entire gene.

We compared a MeC form (19 MeCs) with a non-MeC form (Fig. 1b and c) of the 32-bp exon 7 p53 fragment. G in codon 248 CpG had the largest rate constant, which was 3-fold larger for the MeC version compared to all-C. Rate constants for reactive G’s in non-CpG codons were 5–8 fold smaller than codon 248 MeCpG. Conformational and hydrophobicity changes in the MeC-p53 fragment revealed by circular dichroism (CD) and molecular modeling combine to increase the binding constant of BPDE at the codon 248 site to greatly facilitate the rate of the S_N_2 coupling reaction in line with the preceding non-covalent binding pathway.

## Results and Discussion

**Figure 1:**
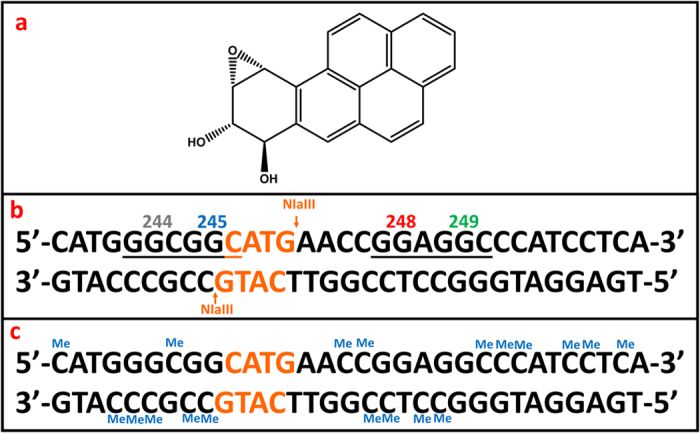
(**a**) Structure of (+)-anti-benzo[a]pyrene-7,8-dihydrodiol-9,10-epoxide (BPDE); (**b**) Exon 7 32 bp fragment of p53 gene with major hot spots 244, 245, 248 and 249 labeled grey, blue, red and green. Restriction enzyme cleavage sites CATG in orange (**b**) all-C version; (**c**) MeC version, all Me except C in restriction enzyme cleavage site.

**Figure 2:**
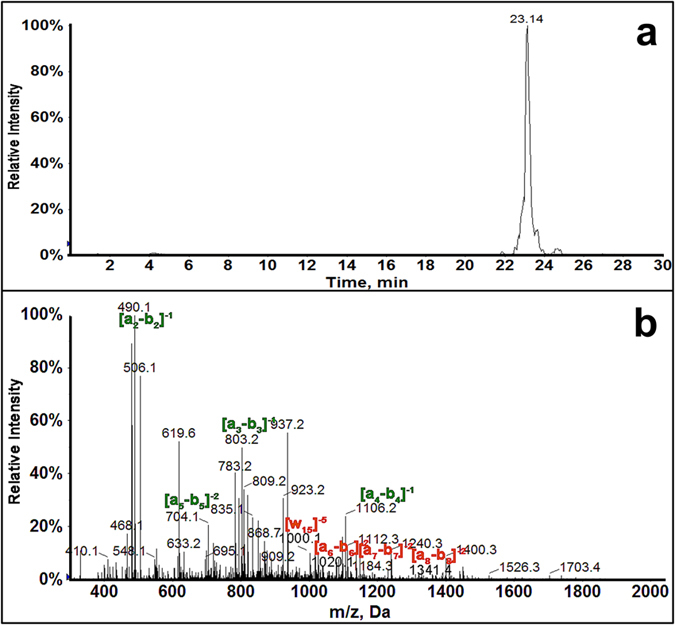
LC-MS/MS of sub-Fragment 2 of methylated version of 32 base pair exon 7 fragment. (**a**) Extracted ion chromatogram of sub-Fragment 2, m/z 1038.5 with z = −6. (**b**) MS/MS spectra of 1038.5 m/z ion eluting at 23.14 min.

BPDE was reacted with an exon 7 fragment in solution, the BPDE-adducted oligonucleotide is cut by restriction enzyme NlAIII, then denatured by heat to obtain 4 single strand (ss) fragments (Fig. 1b and c) that were analyzed by LC-MS/MS sequencing. MS/MS spectra of undamaged fragments were compared to that of singly adducted fragments to identify BPDE-adducted strands (Table S1, Supplementary Information (SI) file). The extracted ion chromatogram (XIC) for singly adducted methylated ss-Fragment 2, m/z 1038.5 z = −6 (Fig. 2a) shows a single peak, indicating only one singly-adducted fragment 2 for the MeC exon 7 fragment. The MS/MS spectrum (Fig. 2b) shows a_n_-b_n_ values similar to the undamaged MeC exon 7 up to a_5_-b_5_ and an increase in m/z from a_6_-b_6_ and above indicating adduction on the 5^th^ base. (AA^Me^C^Me^CG*GAGG^Me^C^Me^C^Me^CAT^Me^C^Me^CT^Me^CA, * = adduction site). This is confirmed by w ions similar to unreacted MeC fragment up to w_14_ with an increase in w_15_ (Table S2, SI file). Similar MS/MS spectra were analyzed for Fragment 1. (see SI. MS/MS spectra indicated two positional isomers for singly adducted fragment 1: (^Me^CATGG*G^Me^CGGCATG) and (^Me^CATG*GG^Me^CGGCATG).

MS/MS spectra obtained from the non-MeC version were similar to those reported in our previous study^20^. Codons selectively reacted with BPDE in MeC and C versions of the exon 7 fragment were 248, 244 and 243. As a control, BPDE was reacted with 32 bp exon 7 fragment of p53 having only one MeC adjacent to codon 245 while all others were un-methylated. No change in reaction selectivity was found.

### Kinetics

Multiple reaction monitoring (MRM) was then used to quantify BPDE adduction at each reactive codon vs. reaction time. Transitions were selected specific to the ss-fragment monitored (Table S4, SI file). For example, transition 1085.4 → 650.0 for singly adducted MeC fragment 1, and 1009.9 → 650.0 for unadducted fragment 1. Here 1085.4 and 1009.9 are m/z of precursor ions of single adducted fragment 1 and unadducted fragment 1, respectively, with charge −4. M/z 650.0 represents the product ion of precursor 1085.4 (Figure S1b and c) which is also the major transition for the unadducted precursor ion, m/z 1009.9.

**Figure 3:**
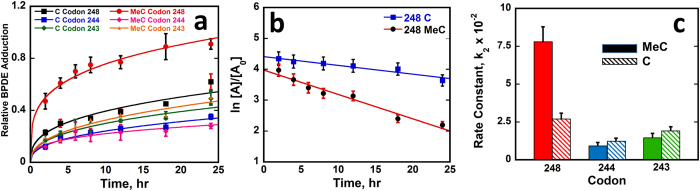
Kinetic study of BPDE adduction on MeC and all-C 32 bp p53 exon 7 gene fragments. (**a**) Relative amount of BPDE adducted to guanine within codons 248, 244 and 243. (**b**) Rate plots showing natural log of relative amount of undamaged oligo fragments (ln [*A*]*/*[*A*_*0*_]) vs time. (**c**) Bar graph showing comparative rate constants k_2_ (s^−1^M^−1^) calculated from the slope of rate plots for BPDE adduction. Error bars represent SD for n = 3.

The relative amount of BPDE adduction was measured as ratio of peak area for XIC of adducted fragment to total peak area of the corresponding adducted + unadducted fragment. Relative amounts of BPDE adduction were plotted vs. time for G’s in codons 248, 244 and 243 (Fig. 3a).

Expressions in eq (1) define k_1_ as the pseudo-first order rate constant, and k_2_ as the second order rate constant, where C_o_ is initial amount of unreacted exon 7 fragment and C the amount unreacted at time t. Linear plots that fit eq 1 were obtained (Fig. 3b), as shown for codon 248 for MeC and all C exon 7 fragments. We obtained k_1_ from the slopes, and k_2_ from k_1_ (equation 1). Plots for the other codons are in Figure S2, SI file.


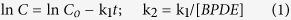


Kinetic results show that k_2_ for BPDE adduction on codon 248 CpG is nearly 3-fold larger for the MeC fragment vs. C-only (Fig. 3c, Table 1). Rate constants for non-CpG adduct at codons 244 and 243, are ~20% smaller for MeC vs C-only, but differences are not statistically significant. The k_2_ ratio of the MeC oligonucleotide for codons 248/244 is ~9, close to ratio of mutational frequencies in the p53 gene^2^. For MeC exon 7 (Table 1), codon 248/243 k_2_ ratio is 5.3 while the mutation ratio is a bit larger at 24. These results demonstrate the influence of MeC on CpG sites like codon 248 in the p53 gene to greatly increase reaction rates of MeCpG sites. Results show negligible influence of MeC on reactive non-CpG guanines.

### Structural Analysis

**Figure 4:**
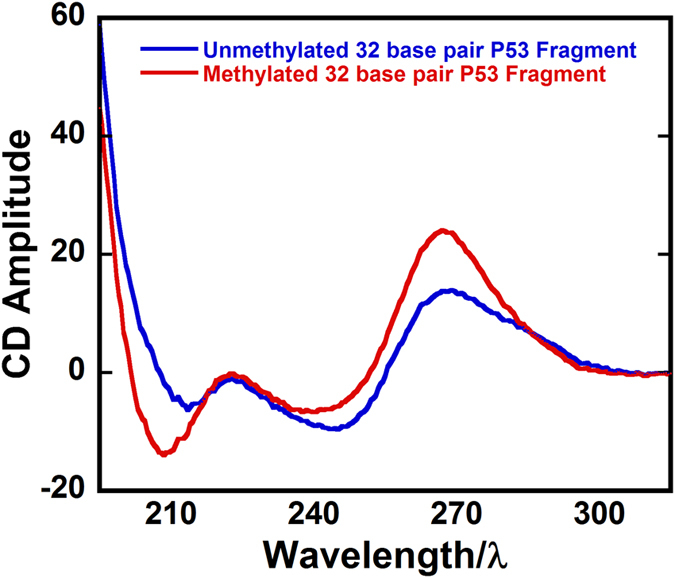
Circular dichroism spectra of the full MeC and all-C versions of 32-bp exon 7 p53 fragments in the pH 7.4 reaction buffer.

Additional studies were aimed at molecular interpretation of the kinetics. Circular dichroism (CD) spectra of full MeC and C-only versions of the exon 7 fragments (Fig. 4), suggest different conformations (Figures S3, S4, SI for full analysis). The MeC exon 7 has an intense negative CD peak at 210 nm and an intense positive peak near 270 nm similar to a pure A-DNA structure^21^, but also a minimum near 245 nm characteristic of B-DNA. For the non-MeC exon 7, the first minimum is shifted to longer wavelength and is weaker, and a maximum at 265 nm is broad, with a shoulder at ~285 nm more characteristic of B DNA^21^ (Fig. 4). We interpret both CD spectra in terms of mixed A-B DNA structures, with MeC’s driving structure toward the A.

### Molecular Modeling

**Figure 5:**
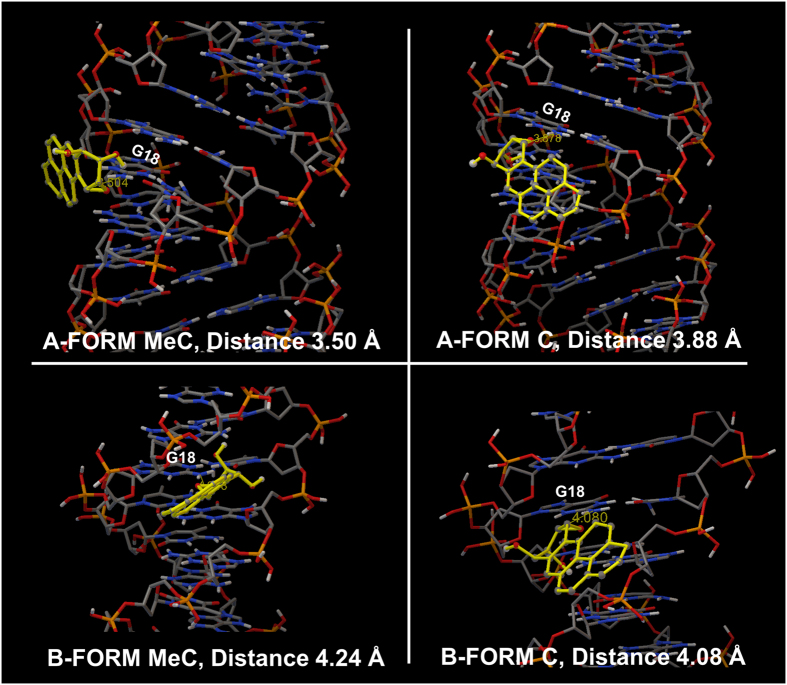
Models of BPDE docked close to reactive guanine in codon 248 in A and B forms of the 32 bp exon 7 p53 fragment in MeC and C versions. Distance is between exocyclic amine of reactive G and epoxide carbon in Å. (Water is removed for clarity; solvated models with water in SI.).

A and B forms of Me-C and C versions of exon 7 were constructed and molecular modeling was done using Autodock software. Structures were solvated with water and docked with the most reactive isomer (+) anti-BPDE. BPDE conformations at optimal docking sites were in the minor groove close to codon 248 for conformations with the most negative binding free energy. Conformations with distances between reactive exocyclic amine of G in codon 248 and the epoxide carbon of BPDE less 4.5 Å were considered, due to probability of subsequently forming covalent bonds. Optimal binding of BPDE to guanine in codon 248 (Fig. 5) gave binding free energies (∆G_b_) for B and A conformations (Table 2) which were used to calculate binding constants (K_b_) from *K*_*b*_ = −*∆G*_*b*_*/RT*, where R is the ideal gas constant and T is in Kelvin. Larger K_b_’s were found for MeC versions in both A and B form of DNA compared to all-C counterparts. For conformations approximating experimental ones, MeC A-form had 5-fold higher K_b_ than all-C A-form. Smaller interatomic distances of reactive atoms were found in A-form of DNA (Fig. 5, Table 2) indicating better accessibility for BPDE, with the smallest distance for the MeC A-form. Docking studies were also done with (-) anti BPDE (SI file), which gave qualitatively similar results but less dramatic K_b_ differences. As a control, we modeled BPDE binding to ds-poly(dG-dC).(dG-dC) oligonucleotides with all MeCs and all C, and found 5-fold larger K_b_ for MeC version similar to experimental measurements^18^. Thus, modeling of the MeC exon 7 fragment as an A DNA structure and the all-C version closer to B DNA agreed well with a pathway featuring preceding non-covalent binding of BPDE in the minor groove near codon 248 that “sets up” subsequent fast S_N_2 covalent coupling.

It’s very likely that stronger binding to the A DNA-like structure of the MeC exon 7 is also influenced considerably by hydrophobic interactions that increase for A DNA-like MeC oligonucleotides. An indirect indication of this effect was found in preliminary modeling studies without water, in which similar trends were found in K_b_ for systems in Fig. 5, but K_b_ differences between A MeC and B all-C forms were much smaller. We thus attribute a part of the increases in K_b_ (Table 2) for both the A and B forms of the MeC p53 fragments to the hydrophobic influence on water structure that tends to increase affinity of BPDE for the codon 248 minor groove.

Our findings of B-like to A-like structural changes for conversion from the C → MeC exon 7 duplexes are consistent with earlier literature^22^,^23^. Crystalized oligonucleotides are predominantly B DNA, but can transition to dehydrated A-forms upon methylation when rich in CG regions, and intermediate structures between A and B have been crystallized^24^,^25^. The A form has a wider minor groove that provides better accessibility for BPDE^21^. This enables a shorter distance for the reactive exocyclic amine of G to the epoxide carbon of BPDE in A-form than in B form (Table 2). Earlier computations showed that epigenetic modifications alter the structure of the DNA making sites of adduction more accessible^26^,^27^,^28^,^29^.

## Conclusions

Methods utilized above provide a straightforward approach to directly study kinetics of gene damage reactions. Results suggest that methylcytosines, which predominate in tumor suppressor genes^4^, and influence the kinetics of S_N_2 reactions with BPDE mainly at CpG sites of tumor suppressor genes. In the p53 exon 7 fragment studied, codons 248, 244 and 243 were the reactive sites for MeC and all-C versions. Codon 248, the featuring CpG, gave the fastest reaction with the MeC fragment reacting 3-fold faster than the non-MeC version (Table 1). CD spectra and computation modeling uncovered a change in conformation from a mixed A-B to a more A-like duplex structure that drives free-energy for noncovalent binding of BPDE in the codon 248 region more negative for the MeC version, due to better access to the minor groove site and increased hydrophobicity. The resulting larger K_b_ most likely lowers activation free energy to contribute significantly to the faster kinetics of S_N_2 coupling of BPDE to MeCpG in codon 248. The structural change does not significantly influence non-CpG codons 244 and 243 that have similar kinetics for MeC and C versions. The hydrolysis-free methodology used here to measure direct kinetics of damage by metabolites to oligonucleotides longer than 20 bp is applicable to correlate gene damage sites for drug and pollutant metabolites with mutation sites. We speculate that these longer nucleotides are more amenable than shorter fragments to uncover the influence of important structural changes relevant to the reactivity of the entire gene.

Methodology described is directly adaptable to other chemicals and other tumor suppressor gene fragments to investigate kinetics of their DNA damage reactions. Molecular dynamics modeling can be used as an auxiliary tool to gain a more complete assessment of the chemistry of the associated reaction events. This quantitative methodology can be adapted to multiple chemicals and multiple exons across multiple tumor suppressor genes to expanding knowledge of genotoxicity chemistry pathways in relation to organ specificity of carcinogenesis.

## Materials and Methods

### Chemicals and Reagents

Benzo[a]pyrene-r-7,t-8-dihydrodiol-t-9,10-epoxide (±) (anti) (anti-BPDE) was from National Cancer Institute Chemical Carcinogen Reference Standard Repository. Triethylammonium bicarbonate (1.0 M, pH 8.6), HPLC-grade methanol, acetonitrile and water were obtained from Sigma Aldrich. Custom made 32 base oligonucleotide fragments exon 7 p53 that were methylated on all C’s (MeC) or had no MeCs were from Sigma Aldrich. All oligonucleotides were HPLC purified and the mass of non-MeC forward fragment is 9820, reverse fragment is 9833 and for the MeC version, forward strand has a mass of 9961 indicating the presence of 10 MeC and for reverse strand 9960 indicating the presence of 9 MeC. Restriction enzyme NlaIII was from New England Biolabs.

### Caution

Benzo[a]pyrene-r-7,t-8-dihydrodiol-t-9,10-epoxide (±) (anti) (anti BPDE) is a known chemical carcinogen. Protective measure like wearing gloves and protective eyewear were taken while doing experiment. All experiments were performed in a closed hood.

### Reaction of Exon 7 Fragments with BPDE

100 μg (~5 nmol) of ds-32 base pair exon 7 fragment is reacted with 50 nmol of BPDE in a total reaction volume of 150 μL, 10 mM Tris buffer pH 7.4 and 50 mM sodium chloride in the dark controlled at 25 ± 0.5 °C. This reaction was performed at various time intervals 2, 4, 6, 8, 12 and 24 hours for both unmethylated C and fully MeC versions of the ds-32 base pair exon 7 fragment. Reactions were stopped by adding cold acetonitrile and excess BPDE was removed using 3000 da molecular weight cut off filters (from EMD Millipore, UFC500396), which allows BPDE to pass through and DNA was collected from the filter as described previously^20^. These ds-32 base pair p53 gene fragments were subjected to restriction enzyme treatment and purification steps before subjected to LC-MS/MS analysis. All LC-MS/MS samples were run in triplicates.

### Circular Dichroism

Circular dichroism experiments on the C and MeC ds-32 base pair exon 7 oligonucleotides were performed on Jasco spectrophotometer (J-710), in 10 mM Tris buffer pH 7.4 and 50 mM sodium chloride. Parameter used within the spectrophotometer include sensitivity of 100mdeg, wavelength range 195 nm to 230 nm with a bandwidth of 5.0 nm.

### Restriction enzyme treatment on ds-32 base pair DNA

Approximately 100 μg of ds-32 base pair was recovered from the reaction mixture and treated with 10 μL (100 units) of NlaIII enzyme, 20 μL of 10X NE buffer (from New England Biolabs) and the volume was made up to 200 μL with water. The reaction mixture was incubated at 37 **°**C for 8 hours. DNA fragments were extracted from restriction enzyme reaction mixture using a previously described protocol using phenol/chloroform/isoamylalcohol, 25/24/1 and chloroform/isoamylalcohol, 24/1 to remove proteins. Briefly mixture of DNA and RE enzymes was vortexed with equal volume of phenol/chloroform/isoamyl alcohol for 15 min followed by centrifugation for 10 min, organic phase discarded and aqueous phase was collected (for 3 times), and then repeated the same process with chloroform/isoamylalcohol (2 times). Finally the obtained DNA fragments from aqueous phase were subjected to desalting using Water’s Oasis HLB cartridges (WAT094226) by solid phase extraction. Briefly cartridges were washed with methanol and water for equilibration followed by sample addition and washing the salts with 5% methanol and elution with 100% methanol. Obtained samples were evaporated in a rotovap, re-dissolved in water and heated and cooled to obtain ss fragments. Stored at −20 °C until use.

### Molecular modeling

A and B form’s of 32-base pair p53 DNA was modeled using make-na software^30^ and modified with cytosines methylated using Maestro software and minimized^31^. Solvated models of these modified oligonucleotides were created using CHIMERA software^32^,^33^, Amber solvation model was used for solvation with a box size of 1 Å to accommodate water molecules. Autodock 4.2.6 was used for docking studies. Prepared biomolecule (Solvated MeC and C 32 base pair exon 7 fragment) were imported into the software. Lamarckian genetic algorithm (LGA) was used in Autodock 4.2.6 to find binding energy between the gene fragments and BPDE. Grid or volume for docking studies were kept constant for all the confirmations and set to be at maximum. Binding energies, binding constants and the distance between the exocyclic amine of the reactive guanine and epoxide carbon of BPDE were calculated^28^. Detailed steps for molecular modeling are given in SI.

## Additional Information

**How to cite this article**: Malla, S. *et al*. Methyl-Cytosine-Driven Structural Changes Enhance Adduction Kinetics of an Exon 7 fragment of the p53 Gene. *Sci. Rep.* **7**, 40890; doi: 10.1038/srep40890 (2017).

**Publisher's note:** Springer Nature remains neutral with regard to jurisdictional claims in published maps and institutional affiliations.

## Supplementary Material

Supplementary Information

## Figures and Tables

**Table 1:** Rate constants k_1_ and k_2_ and ratios for different reactive sites.

Rate Constant	MeC Codon			C Codon		
	248	244	243	248	244	243
k_1_, s^−1^ (x10^6^)	26 ± 3	3.0 ± 0.7	4.8 ± 1.0	9.0 ± 1.3	4.0 ± 0.7	6.3 ± 0.9
k_2_, s^−1^M^−1^ (x10^2^)	7.8 ± 1.0	0.9 ± 0.2	1.4 ± 0.3	2.7 ± 0.4	1.2 ± 0.2	1.9 ± 0.3
Rate Constant Ratios						
**Me-C/C**	**Ratio**	**Me-C/Me-C Codon**			**C-C Codon**	
248	2.9	**248/244**	8.7		**248/244**	2.2
244	0.8	**248/243**	5.4		**248/243**	1.4
243	0.8					

Note. Rate constants k_1_, for codon 244 and 243 in the methylated fragments are not significantly different according to t-test at 95% confidence interval.

**Table 2:** Computed binding free energies, binding constant and distance between exocyclic amine of reactive G in codon 248 and epoxide carbon of BPDE.

DNA	Binding Energy kcal.mol^−1^, ∆G	Binding Constant, K_b,_ M^−1^,	Distance, Å
B Form-C	−3.47	3.48 × 10^2^	4.08
B Form-MeC	−4.10	1.00 × 10^3^	4.24
A Form-C	−3.84	6.58 × 10^2^	3.88
A Form-MeC	−4.80	3.32 × 10^3^	3.50
