# Supplementary material for: Methyl-Cytosine-Driven Structural Changes Enhance Adduction Kinetics of an Exon 7 fragment of the p53 Gene
Source: Sci Rep. 2017 Jan 19;7:40890. doi: 10.1038/srep40890 (PMC5244379; doi:10.1038/srep40890)
Supplement: Supplementary Information [file srep40890-s1.pdf]

Methyl-Cytosine-Driven Structural Changes Enhance Adduction Kinetics of an Exon 7 fragment of the p53 Gene

Spundana Malla, Karteek Kadimisetty, You-Jun Fu, Dharamainder Choudhary, John B. Schenkman and James F. Rusling

SUPPLEMENTARY INFORMATION

TABLE OF CONTENTS

Topic/Item	Page
Experimental Section	
LC-MS/MS conditions	S1
TableS1 Calculated m/z for methylated, unmethylated and adducted fragments	S2
LC-MS/MS data for fragment 2 and fragment 1 of Methylated DNA	S2
Figure S1, Table S2, Table S3, XIC and MS/MS spectra for fragment 1	S3
MRM Transitions selected	S4
Rate plots (Figure S2)	S4
Characteristic Circular dichroism spectra (Figure S3)	S4
CD spectra of MeC and C of 32 base pair exon 7 fragment (Figure S4)	S4
Molecular Modeling (Figure S5)	S5
Table S4, S5 and S6 Molecular modelling data, hydrogen bond, binding energies for 32 base pair exon 7 fragment and polyGC	S6
IARC DATA (Figure S6)	S7

Experimental Section:

LC-MS/MS

Thermo Scientific Ultimate 3000 UPLC with Gemini C-18 column (0.5 mm ID and 3μ particle size and 150mm length) was used. A binary solvent system with 25 mM triethyl ammonium bicarbonate as buffer A and 100 % methanol as solvent B was used. Separation protocol was 0-3 % B (Methanol) for 3 min, then increased gradient from 3 to 27 % B (Methanol) for 24 min, then back to 3 % for 3 min. Between each run a wash is performed with gradient from 3% B isocratic for 2 min followed by 3-80 % B for 24 min followed by equilibrating back to 3 % for 4 min at 10 μL/min. m-Nitrobenzylalcohol was used via syringe pump post column through three way connector to increase the intensity and charge states of oligonucleotide fragments.[1] Absciex Q-Tof and QTRAP 4000 instruments were used in negative mode. Product ion scanning was used for qualitative evaluation of site selective of BPDE-DNA adduction and multiple reaction monitoring (MRM) was used for the kinetic studies at a particular reactive site. For the AB Sciex QSTAR, -4500 ion spray voltage, -130 declustering potential and 300°C temperature was used with collision energy from -42 to -50 eV for MS/MS analysis. MRM on the QTRAP 4000 was done with ion spray voltage of -4500, curtain gas at 45 units, -115 V declustering potential and collision energy varying from -60 to -80 eV was used. All samples were run in triplicate.

The protocol for analyzing the 32 base pair p53 exon 7 fragments was described previously. Briefly, restriction enzyme NlaIII is used to cut the 32 bp DNA 4 smaller fragments for LC-MS/MS analysis. Product ion scan or collision induced dissociation (CID) was used to sequence the oligonucleotides. CID of oligonucleotides gives characteristic a_n-b_n and w_n ions.[2,3] Comparing the MS/MS spectrum of un-adducted oligonucleotides with that of adducted oligonucleotides determines the exact site of adduction. Fragments obtained after restriction enzyme treatment for methylated 32 base pair DNA fragment are shown in scheme S1.

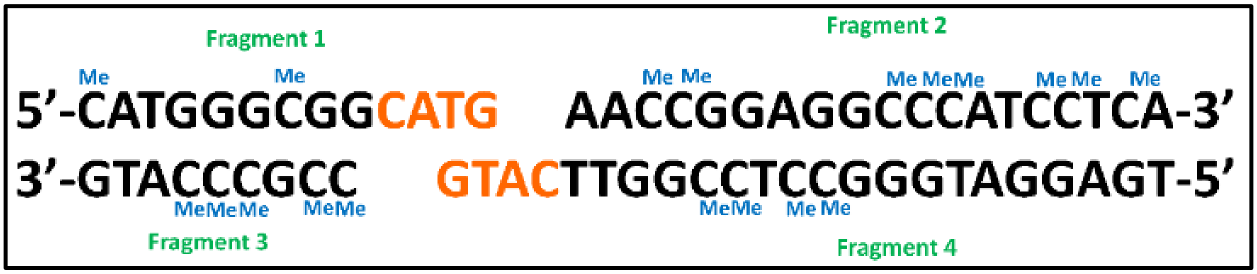

Scheme S1. The four smaller fragments produced after restriction enzyme and heat treatment for 32 base pair MeC-exon 7 fragment.

Table S1: Calculated m/z for fragment 1, fragment 2, fragment 3 and fragment 4 for unadducted and singly adducted (increase in mass of 302.323, BPDE) of methylated, unmethylated versions of 32 base pair exon 7 fragment. Unmodified cytosine in methylated version shown in red. m/z obtained for each fragment is highlighted in bold.

CATGGGCGGCATG Fragment 1				
**z**	**C**	**C Add**	**MeC**	**MeC Add**
1	4014.6	4316.9	4042.6	4344.9
2	2006.8	2158.0	2020.8	2171.9
3	1337.5	1438.3	1346.9	1447.6
4	**1002.9**	**1078.5**	**1009.9**	**1085.5**
5	802.1	862.6	807.7	868.2
6	668.3	718.6	672.9	723.3
7	572.7	615.8	576.7	619.8

AACCGGAGGCCCATCCTCA, Fragment 2				
**z**	**C**	**C Add**	**MeC**	**MeC Add**
1	5821.8	6124.1	5933.8	6236.1
2	2910.4	3061.5	2966.4	3117.5
3	1939.9	2040.7	1977.2	2078.0
4	1454.7	1530.3	1482.7	1558.3
5	1163.547	1224.012	1185.9	1246.4
6	**969.4**	**1019.8**	**988.1**	**1038.5**
7	830.8	874.006	846.8	890.

CCGCCCATG, Fragment 3				
**Z**	**C**	**C Add**	**MeC**	**MeC Add**
1	2738.8	3041.0	2808.7	3111.0
2	1368.9	1520.0	1403.8	1555.0
3	**912.3**	**1013.0**	**935.6**	**1036.3**
4	683.9	759.5	701.4	777.0
5	546.9	607.4	560.9	621.4
6	455.6	506.0	467.3	517.7
7	390.4	433.6	400.4	443.6
8	341.5	379.2	350.2	388.0

TGAGGATGGGCCTCCGGTTCATG, Fragment 4				
**Z**	**C**	**C Add**	**MeC**	**MeC Add**
1	7110.6	7413.0	7166.7	7469.0
2	3554.8	3706.0	3582.8	3734.0
3	2369.5	2470.3	2388.2	2489.0
4	1776.9	1852.5	1790.9	1866.5
5	1421.3	1481.8	1432.5	1493.0
6	**1184.3**	**1234.6**	**1193.6**	**1243.9**
7	1014.9	1058.1	1022.9	1066.1
8	887.9	925.7	894.9	932.7

Table S2: MS/MS fragment ions (a_n-b_n and w_n ions) for fragment 2 obtained. Increase in m/z for a_n-b_n and w_n ions indicating BPDE adduction shown in red.

Ion	NonMeC Fragment 2	MeC Fragment 2
a_2-b_2	$[490.1]^{-1}$	$[490.1]^{-1}$
a_3-b_3	$[803.2]^{-1}$	$[803.2]^{-1}$
a_4-b_4	$[1106.2]^{-1}$	$[1106.2]^{-1}$
a_5-b_5	$[704.2]^{-2}$	$[704.2]^{-2}$
a_6-b_6	$[869.0]^{-2}$	$[1020.2]^{-2}$
a_7-b_7	$[1033.6]^{-2}$	$[1184.7]^{-2}$
a_8-b_8	$[1190.2]^{-2}$	$[1341.4]^{-2}$
w_{13}	$[1009.8]^{-4}$	$[1009.8]^{-4}$
w_{14}	$[1092.2]^{-4}$	$[1092.2]^{-4}$
w_{15}	$[939.4]^{-4}$	$[1000.1]^{-5}$
w_{16}	n/d	$[1060.4]^{-5}$
w_{17}	n/d	$[1404.6]^{-4}$

Table S3: MS/MS fragment ions (a_n-b_n and w_n ions) for fragment 1 obtained. Increase in m/z for a_n-b_n and w_n ions indicating BPDE adduction shown in red.

Ion	NonMeC Fragment 1	MeC-Frag1 Peak 1	MeC-Frag1 Peak II
a_2-b_2	$[400.1]^{-1}$	$[400.1]^{-1}$	$[400.1]^{-1}$
a_3-b_3	$[713.5]^{-1}$	$[713.5]^{-1}$	$[713.5]^{-1}$
a_4-b_4	$[1017.3]^{-1}$	$[1017.3]^{-1}$	$[1017.3]^{-1}$
a_5-b_5	$[1346.4]^{-1}$	$[1346.4]^{-1}$	$[1649.4]^{-1}$
a_6-b_6	$[837.2]^{-2}$	$[988.2]^{-2}$	$[988.2]^{-2}$
a_7-b_7	$[1002.1]^{-2}$	$[1153.7]^{-2}$	$[1153.7]^{-2}$
a_8-b_8	$[768.8]^{-3}$	$[869.5]^{-3}$	$[869.5]^{-3}$
w_2	$[346.1]^{-1}$	$[346.1]^{-1}$	$[346.1]^{-1}$
w_2	$[650.1]^{-1}$	$[650.1]^{-1}$	$[650.1]^{-1}$
w_3	$[963.2]^{-1}$	$[963.2]^{-1}$	$[963.2]^{-1}$
w_4	$[1252.3]^{-1}$	$[1252.3]^{-1}$	$[1252.3]^{-1}$
w_5	$[790.2]^{-2}$	$[790.2]^{-2}$	$[790.2]^{-2}$
w_6	$[954.7]^{-2}$	$[954.7]^{-2}$	$[954.7]^{-2}$
w_7	$[1106.3]^{-2}$	$[1106.3]^{-2}$	$[1106.3]^{-2}$
w_8	$[847.2]^{-3}$	$[847.2]^{-3}$	$[847.2]^{-3}$
w_9	$[955.6]^{-3}$	$[1058.2]^{-3}$	$[955.6]^{-3}$
w_{10}	$[1066.6]^{-3}$	n/d	$[875.1]^{-4}$
w_{11}	$[1168.1]^{-3}$	[951.6]	n/d

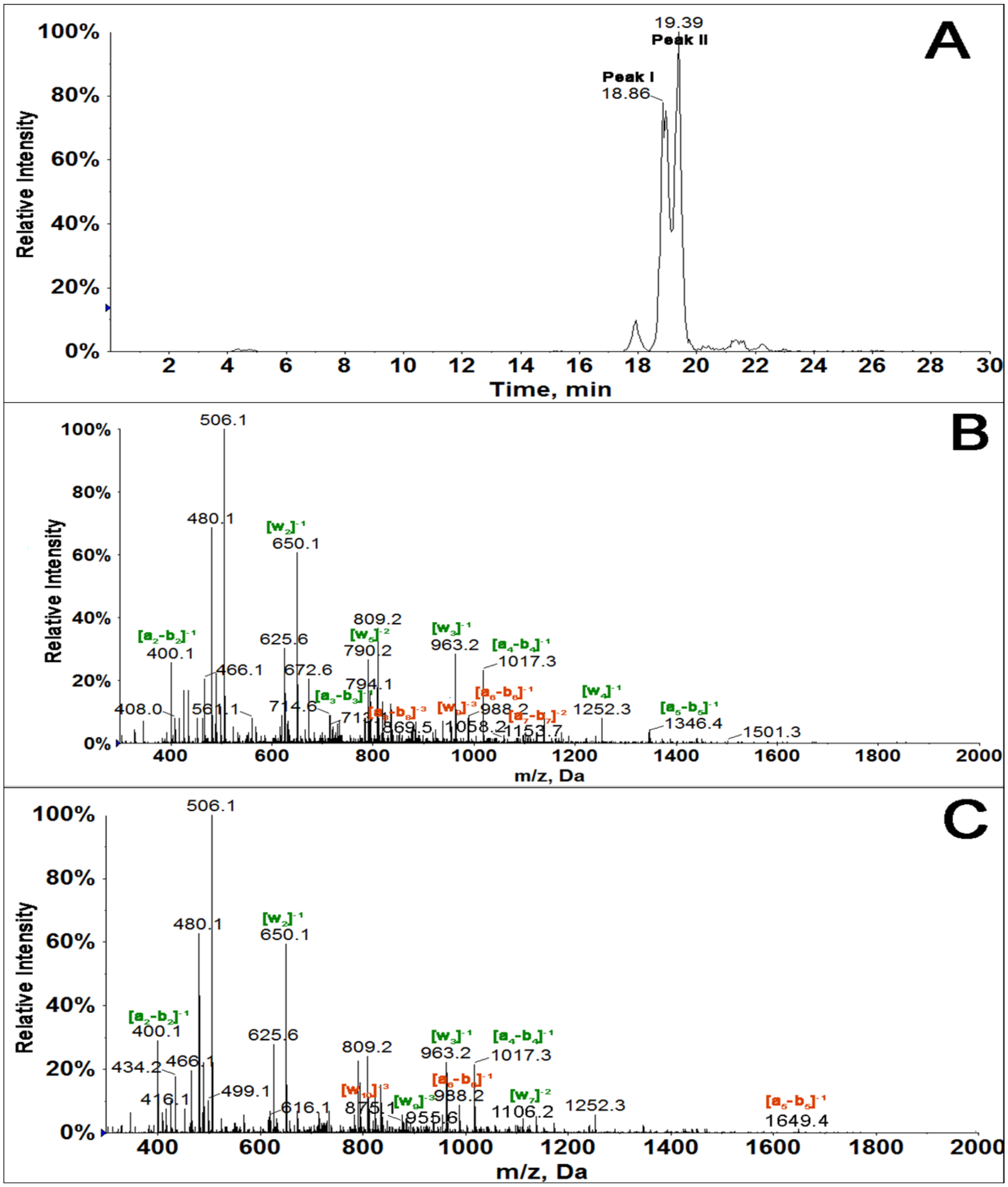

Figure S1. LC-MS/MS of Fragment 1 of MeC 32 base pair exon 7 fragment. A) Extracted ion chromatogram of Fragment 1, m/Z 1085.4 with a charge of -4 B) MS/MS spectra of 1085.4 m/z fragment ion eluting at 18.86 min (Peak I) C) B) MS/MS spectra of 1085.4 m/z fragment ion eluting at 19.39 min (Peak II).

The XIC of Fragment 1, m/z 1085.4 with charge -4 of MeC exon 7 fragment (Figure S1A) gave two major peaks consistent with two positional isomers of singly adducted fragment 1. The MS/MS spectra of the peak eluting at 18.9 min (Figure S1B) show a_n-b_n values similar to the unadducted fragment up to a_5-b_5 and increase in m/z at a_6-b_6, supported by increase in w_n ions from w_9 indicate the adduction (*) at the 6th base (^{Me}CATGG*G^{Me}CGGCATG). The MS/MS spectra of fragment 1 eluting at 19.4 minutes (Figure S1C) show a_n-b_n values similar to unadducted fragment 1 up to a_4-b_4 and increase in a_5-b_5

supported by increase in w_n ions from w_{10} confirming adduction at the 4th base (^{Me}CATG*GG^{Me}CGGCATG) (Table S3, SI file).

Table S4: MRM Transition selected for quantitation of methylated, unmethylated and single adducted version of 32 base pair exon 7 fragment of 53 gene.

Un Methylated	Methylated
1048->383 = Internal Standard	1048->383 = Internal Standard
1078.5->650 = Adducted Fragment 1	1085->650 = Adducted Fragment 1
1003->650 = Fragment 1	1009.9->650 = Fragment 1
1224>923 = Adducted Fragment 2	890->803 = Adducted Fragment 2
1163.5->923 = Fragment 2	846.8->803 = Fragment 2
1184.3->730 = Adducted Fragment 3	1066->650 = Adducted Fragment 3
1234.6->730 = Fragment 3	1022.9->650 = Fragment 3
1013->755 = Adducted Fragment 4	1036->650 = Adducted Fragment 4
912->755 = Fragment 4	935->650 = Fragment 4

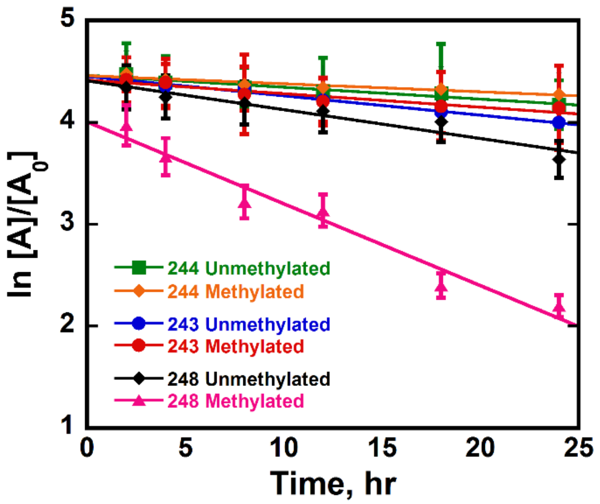

Figure S2. Pseudo-first order rate plots showing the natural log of relative amount of undamaged fragment vs time for all reactive codon, 248, 244 and 243 in both MeC and all-C versions of 32 base pair Exon 7 fragment.

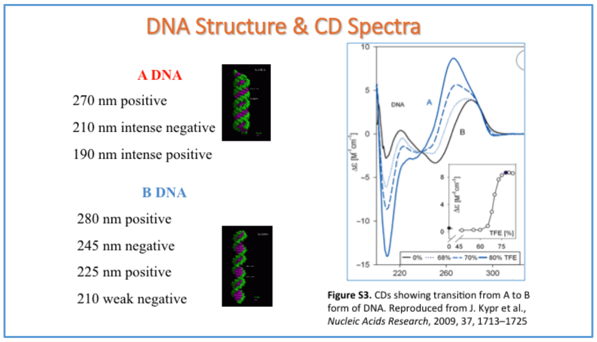

Figure S3. CDs showing transition from A to B form of DNA. Reproduced from J. Kypr et al., *Nucleic Acids Research*, 2009, 37, 1713–1725

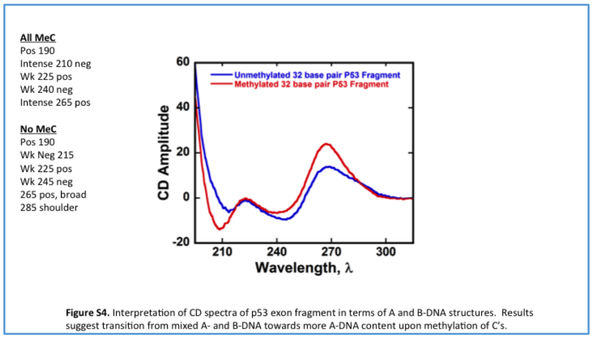

Figure S4. Interpretation of CD spectra of p53 exon fragment in terms of A and B-DNA structures. Results suggest transition from mixed A- and B-DNA towards more A-DNA content upon methylation of C's.

Molecular modeling

A and B form's of 32-base pair P53 DNA was modelled using make-na software[4] and modified with cytosines methylated using Maestro software and minimized.[5] Solvated models of these modified olignucloetides were created using CHIMERA software.[6,7] Amber solvation model was used for solvation with a box size of 1Å to accommodate water molecules.

Autodock 4.2.6 was used for docking studies. Prepared biomolecule (Solvated MeC and C 32 base pair exon 7 fragment) were imported into the software. Lamarckian genetic algorithm (LGA) was used in Autodock 4.2.6 to find binding energy between the gene fragments and BPDE. Grid or volume for docking studies were kept constant for all the confirmations and set to be at maximum (126X x 126Y x 126Z dimensions). Binding energies, binding constants and the distance between the exocyclic amine of the reactive guanine and epoxide carbon of BPDE were calculated.[8]

Procedure for Docking

1. Import the biomolecule
2. Add Hydrogens to the biomolecule
3. Compute gasteiger charges
4. Now input the Ligand
5. Save the output format of the ligand to be in PDBQT (autodock suitable format)
6. Preparation for grid
    a. Choose Macromolcule (32 bp DNA)
    b. Save as PDBQT
    c. Choose ligand (from set map types)
    d. Set grid size (maximum grid size used for our study 126 X 126 X 126).
    e. Save out put file as .gpf (grid format for autodock).
    f. Run autogrid from run option.
7. Docking
    a. Select macromolecule and ligand similar to above from docking menu.
    b. Search Papparmeters given as # of GA runs to be 100, population size 150, maximum evaluations 250000, maximumber of generations 27000 and other factors kept default. Accept the parameters.
    c. Docking parameter kept default.
    d. Output saved as default.
    e. Run autodock from run menu.

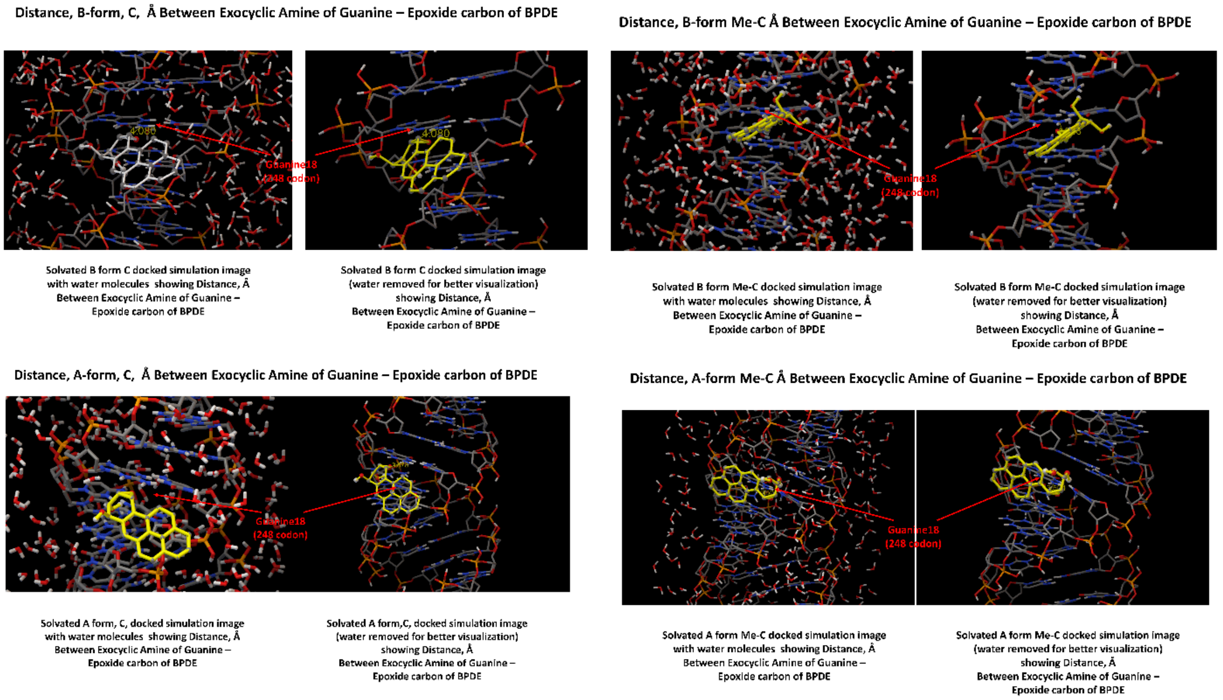

Figure S5. Autodock-simulated images showing BPDE docked close to reactive guanine in codon 248 in A and B forms of the 32 bp exon 7 p53 in both MeC and C versions. Distance between exocyclic amine of reactive G and epoxide carbon shown in Å. Optimal docking structures were chosen as those giving the most negative computed binding free energy,

Table S4. Hydrogen bonds between BPDE and surrounding nucleobases and water molecules along with their bond length.

DNA	# of H Bonds	Type	Bond Length
**B Form-C**	2	Epoxide "O" of BPDE and H on exocyclic amine of reactive guanine	2.05 Å
		BPDE and surrounding water molecules	2.18 Å
**B Form-MeC**	2	Epoxide "O" of BPDE and H on exocyclic amine of reactive guanine	2.20 Å
		Hydroxyl "O" of BPDE and H on exocyclic amine of guanine adjacent to reactive guanine.	1.79 Å
**A Form-C**	2	Epoxide "O" of BPDE and H on exocyclic amine of reactive guanine	1.72 Å
		Hydroxyl "O" of BPDE and H on exocyclic amine of guanine on complimentary strand.	1.70 Å
**A Form-MeC**	1	Hydroxyl H of BPDE and Oxygen on complimentary cytosine	1.94 Å

Table S5. Binding energies, binding constant and distance between exocyclic amine of reactive guanine in codon 248 and epoxide carbon of (-)anti-BPDE.

DNA	Binding Energy kcal/mol, ∆G	Binding Constant M^{-1}, K_b	Distance Å
**B Form-C**	-3.64	4.67×10^2	4.12
**B Form-MeC**	-3.77	5.78×10^2	3.86
**A Form-C**	-4.34	1.52×10^3	3.93
**A Form-MeC**	-4.62	2.43×10^3	3.91

Table S6. Binding energies, binding constant and distance between exocyclic amine of reactive guanine in polyGC and epoxide carbon of BPDE,

DNA	Binding Energy kcal/mol, ∆G	Binding Constant M^{-1}, K_b	Distance Å
**B Form-C**	-3.53	1.41×10^2	5.70
**B Form-MeC**	-3.77	5.84×10^2	3.83
**A Form-C**	-4.01	8.62×10^2	4.21
**A Form-MeC**	-4.43	1.77×10^3	3.11

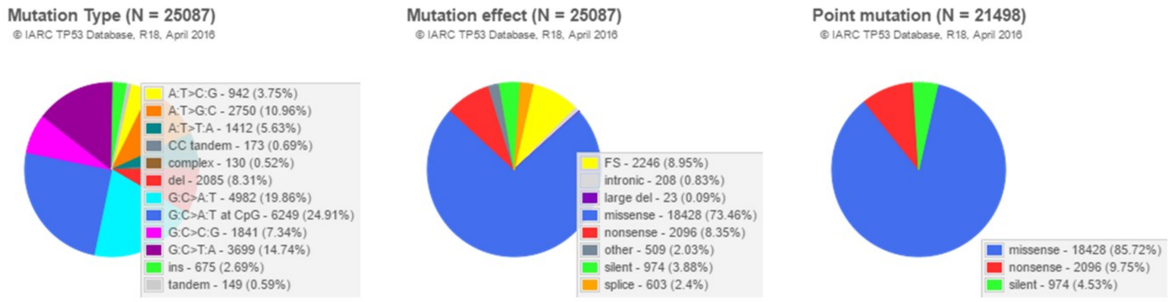

Figure S6. International agency for research on cancer data obtained from P53 data base version R18 based on Pie charts obtained for Mutation type, mutation effect an point mutations.

References

1. Brahim, B., Alves, S., Cole, R. B. & Tabet, J. Charge enhancement of single-stranded dna in negative electrospray ionization using the supercharging reagent meta-nitrobenzyl alcohol. *J. Am. Soc. Mass Spectrom.* **24**, 1988-1996 (2013).
2. Ehrich, M. *et al*. Quantitative high-throughput analysis of DNA methylation patterns by base-specific cleavage and mass spectrometry. *Proc. Natl. Acad. Sci. U. S. A.* **102**, 15785-15790 (2005).
3. Murray, K. K. DNA sequencing by mass spectrometry. *Journal of mass spectrometry* **31**, 1203-1215 (1996).
4. http://structure.usc.edu/make-na/server.html last accessed 09/14/2016
5. Schrodinger, L., maestro, New York, NY, (2006).
6. Pettersen, E. F. *et al*. UCSF Chimera—a visualization system for exploratory research and analysis. *Journal of computational chemistry* **25**, 1605-1612 (2004).
7. http://www.rbvi.ucsf.edu/chimera/ last accessed 09/15/16
8. Morris, G. M. *et al*. AutoDock4 and AutoDockTools4: Automated docking with selective receptor flexibility. *Journal of computational chemistry* **30**, 2785-2791 (2009).
